# Disparities in scientific research activity between doctors and nurses working in the Peruvian health care system: Analysis of a nationally representative sample

**DOI:** 10.1371/journal.pone.0273031

**Published:** 2022-09-02

**Authors:** Angélica Vergara-Mejía, Roberto Niño-Garcia, Ludwing Zeta-Solis, Percy Soto-Becerra, Ali Al-kassab-Córdova, Reneé Pereyra-Elías, Báltica Cabieses, Edward Mezones-Holguin

**Affiliations:** 1 Universidad Nacional Hemilio Valdizán, Escuela de Postgrado, Huánuco, Perúa; 2 Seguro Social en Salud (EsSalud), Hospital Nacional Guillermo Almenara Irigoyen, Lima, Peru; 3 Universidad Nacional de Piura, Facultad de Ciencias de la Salud, Sociedad Científica de Estudiantes de Medicina de la Universidad Nacional de Piura (SOCIEMUNP), Piura, Peru; 4 Universidad San Ignacio de Loyola, Centro de Excelencia en Investigaciones Económicas y Sociales en Salud, Lima, Peru; 5 Epi-gnosis Solutions, Piura, Peru; 6 Nuffield Department of Population Health, University of Oxford, National Perinatal Epidemiology Unit, Oxford, United Kingdom; 7 Facultad de Ciencias de la Salud, Universidad Peruana de Ciencias Aplicadas, Lima, Peru; 8 Facultad de Medicina Clínica Alemana, Universidad del Desarrollo, Programa de Estudios Sociales en Salud-Instituto de Ciencias e Innovación en Salud, Santiago de Chile, Chile; Azienda USL - IRCCS di Reggio Emilia: Azienda Unita Sanitaria Locale - IRCCS Tecnologie Avanzate e Modelli Assistenziali in Oncologia di Reggio Emilia, ITALY

## Abstract

**Aim:**

To evaluate disparities in the frequency of scientific activity between medical doctors and nurses in Peru.

**Methods:**

We carried out a secondary data analysis of the National Health Services Users’ Satisfaction Survey (ENSUSALUD), 2016. This nationally representative survey evaluates doctors and nurses working in clinical settings. We defined scientific activity as i) having published an original article (journal indexed in Web of Science, Scopus or Medline); and ii) having authored an abstract in a national or international conference. We estimated crude and adjusted disparities prevalence ratios (aDPR) and 95% confidence intervals (95%CI).

**Results:**

We included 2025 doctors and 2877 nurses in the analysis; 71% of doctors doctor were male, and 93% of nurses were female (p<0.001). Among doctors, 13.9% had published an article, and 8.4% presented an abstract at a conference in the last two years, while these proportions were 0.6% and 2.5% for nurses, respectively. The adjusted models showed that doctors, when compared to nurses, were approximately 27 times likely to have published a paper (aDPR = 27.86; 95% CI 10.46 to 74.19) and twice as likely to have authored a conference abstract (aDPR = 2.51; 95% CI 1.39 to 4.53).

**Conclusions:**

There are important disparities in scientific activity between doctors and nurses working in clinical settings in Peru. Disparities are more significant for article publication than for authoring in conference abstracts. We suggest public policies that promote research dissemination between health professionals, with emphasis on nurses.

## Introduction

Developing countries, such as Peru, have severe deficiencies in biomedical research, even though this is crucial for improving health care systems [1]. By conducting research, the health workforce plays an essential role in proposing solutions to problems within the health care sector [2]. In this context, medical and nursing professionals constitute the highest proportion of personnel in the health care workforce worldwide, mainly due to their crucial role in the continuity of health care and the ability to provide the minimum necessary support in a basic health care team [3]. Therefore, doctors and nurses have a valuable opportunity to generate evidence and inform decision-making for health care improvement and innovation [4–7]. Subsequently, scientific research activity among doctors and nurses is a growing topic of interest for healthcare systems worldwide (4.8–10).

Scientific research activity is disseminated through two fundamental ways: presentations at conferences and publications in peer-reviewed journals [7–9]. Although both activities are part of a continuous process with publication as the final goal, there is a disparity between the number of investigations carried out, studies presented at conferences and the final number of published papers [10,11]. Our study analyzed both abstract conference presentations and publications in peer-reviewed journals and their differences between doctors and nurses. We hypothesized that doctors might be more engaged in these activities than nurses because of the inequitable distribution of several factors, such as working, training and incentive programs [4,12–14]. Therefore, studying disparities in scientific research activity between physicians and nurses is pertinent to Latin American countries, like Peru.

Numerous factors avert the appropriate development of scientific research in medical personnel in Peru. First, scientific research is a scarcely endorsed effort in a country with a 0.12% GDP expenditure in research in 2015, lower than the average in Latin America, and lower than other nearby countries, such as Chile (0.38%, 2014), Bolivia (2009: 0.16%,), Colombia (2014: 0.20%), Ecuador (2011: 0,34%), Brazil (2013: 1,24%), and Mexico (2014: 0.54%) [15]. Likewise, only 10% of the GDP destined for research corresponded to the area of health sciences [16,17], with fewer incentives for the development of scientific research than developed countries [18]. Second, the Peruvian health care system is complex and fragmented, with a high percentage of professionals simultaneously working in other institutions [19,20]. Third, in Peru, there is no efficient articulation between health service providers, decision makers and human resources for health, three groups that remain relatively isolated from the development of scientific research [21]. Further joint efforts and collaboration is needed among these professionals and institutions. All the factors previously described affect scientific production in the entire country; nonetheless, there are some factors unequally distributed between doctors and nurses that may generate a gap in scientific research activities between them. Some of these factors include training time, university curricula, scientific societies, and academic activities. Hence, it is relevant to identify and quantify disparities in scientific research activities between doctors and nurses who work in the Peruvian health care system. All in all, the inequalities of scientific production hinder the improvement of the Peruvian health care system.

Despite the acknowledged importance of scientific research in the health arena, to the best of our knowledge, there are no studies that evaluate the disparities in scientific research activity between Peruvian doctors and nurses. For the reasons mentioned above, our study aimed to evaluate the disparities in scientific research activity between doctors and nurses working in health care centers in Peru. Our results expose new challenges in these healthcare professions and could serve to inform decision-making in the Peruvian health system.

## Methods

### Data source and study population

We carried out a secondary analysis of the National Healthcare Satisfaction Survey (ENSUSALUD, by its Spanish acronym) from 2016. ENSUSALUD-2016 is a cross-sectional survey of a nationally representative sample of users from Peruvian health facilities [22]. It was conducted annually from 2014, and 2016 is the latest survey conducted so far. ENSUSALUD-2016 was implemented under the authority and funding of the National Health Superintendence (SUSALUD, by acronym in Spanish), Ministry of Health of Peru and was conducted by the National Institute of Statistics and Informatics from April to July 2016. ENSUSALUD-2016 collected data through six questionnaires, each of which was targeted to one specific user of health care system: outpatients; doctors and nurses, users of health insurance offices; users of pharmacies and drugstores; management staff of health facilities; and emergency patients. Detailed information about the design and execution of ENSUSALUD-2016 is available on the official web of SUSALUD (http://portal.susalud.gob.pe/wp-content/uploads/archivo/encuesta-sat-nac/2016/INFORME_FINAL_ENSUSALUD_2016.pdf). This study only analyzed data from the second questionnaire, which was addressed to the doctors and nurses who worked in health facilities from the public and private sectors of the Peruvian Health Care System for at least 12 months at the time of the interview. The data set is freely available in a public repository within the official website of the SUSALUD (http://portal.susalud.gob.pe/wp-content/uploads/archivo/base-de-datos/2016/C2_CAPITULOS%20-%20PROFESIONALES%20MEDICOS%20Y%20ENFERMERAS.sav).

ENSUSALUD-2016 followed a stratified two-stage cluster sample design. Peru is divided into 24 geopolitical regions (GR), and its healthcare system is fragmented into four sectors. Independently of the sector, health facilities are classified according to their complexity level into categories from I-1 (minimum resolutive capacity) to III-E (maximum complexity). The primary sampling unit (PSU) was the health facility of any health sector with a category that reflex an intermediate or more resolutive capacity (I-4, I-4/I-3, II-1, II-2, II-E, III-1, III-2 and III-E). The secondary sampling unit (SSU) was the doctors and nurses who worked at least one year in the health facilities selected for the survey. In the first stage of sampling, PSUs were stratified by GR proportionally to the number of healthcare consults. N health facilities were systematically selected after randomly selecting a starting point within each GR. The second stage involved randomly selecting n SSU per PSU. The survey included sampling weights to compensate for the unequal distribution of the probability of selection among the strata and stages and the losses due to non-response.

In total, 183 PSUs and 5,098 doctors and nurses were surveyed at the national level by ENSUSALUD-2016. This secondary analysis only included those who completed their degree at a Peruvian university and have complete information in the variables included in the models.

### Instruments, variables, and data processing

Questionnaire 2 of ENSUSALUD-2016 was composed of 110 questions designed to obtain information from doctors and nurses about their sociodemographic characteristics, academic and work profile, work-related satisfaction, mental health issues, among others (http://portal.susalud.gob.pe/wp-content/uploads/archivo/encuesta-sat-nac/2016/Cuestionario%202%20-%20Profesionales%20medicos%20y%20enfermeria.pdf).

The outcome variables were the following indicators of scientific research activity: (i) self-report of the publication of at least one original article in a journal indexed in Web of Science (WoS), SCOPUS or Medline (yes/no), (ii) number of articles ever published among doctors or nurses that indicate having ever published in their life, (iii) time from publication of the latest article (years) among doctors or nurses that indicate having ever published in their life, and (iv) participation as an author of an abstract presented at a national/international (or both) conference within the last two years (yes/no). For the published papers, ENSUSALUD-2016 verified that the journal’s name indicated by the participant was found in the corresponding database. Only publications of such databases were included as a proxy for high-quality scientific production. Likewise, those databases are recognized by the Peruvian National Council of Science and Technology (CONYTEC, from Spanish acronym). The primary exposure variable was the self-report of the profession, divided into medical doctors and nurses (the only two professional groups included in ENSUSALUD).

We also included the following variables to control for potentially confounding effect: age (years), sex (male/female), place of residence (Metropolitan Lima/Other regions), marital status (married-cohabiting/single, widowed or divorced), living arrangements (accompanied/alone), University location (Metropolitan Lima/other regions), type of university (private/public), age of onset of professional practice (years), lecturer/professor at university (no/yes) and postgraduate studies (no/yes).

The dataset was downloaded from the official web repository of SUSALUD in SPSS format («*.sav») and converted to Stata v14 format («*.dta») using the library *haven* [23] from the packages collection *tidyverse* [24] in R language and statistical software version 3.6.0 (2019-04-26) [25] for Microsoft Windows 10 Pro x86_64 bits. Reproducible code in R for converting SPPS to Stata format is available in S1 File. Raw dataset (obtained immediately after converting SPSS to Stata format) are available in S2 File. Variables (outcomes, exposures and covariates) analyzed in this study were generated using reproducible code in Stata format («*.do») that is available in S3 File. Tidy dataset (obtained after running the code in S2 File) is available in S4 File.

### Statistical analysis

We described the numerical variables using mean (standard deviation [SD]), and they were compared between both professional groups using the Wald test design-based [26,27]. Categorical variables were reported as relative frequencies (percentages) and compared by a Pearson Chi-squared test adjusted by the sampling design with the second-order correction of Rao and Scott [27,28].

The primary analysis sought to estimate professional disparities in research activities after controlling for potentially confounding. In the disparities of dichotomous outcomes, we used Poisson generalized linear models with a log link function to estimate crude and adjusted disparity prevalence ratios (cDPR and aDPR, respectively), which are statistically identical to prevalence ratios. For numerical outcomes, such as the number of articles published and the time from the last article published, we calculated crude and adjusted disparity mean ratios (cDMR and aDMR, respectively) using negative binomial regression. We parametrized the variance as a function of the mean μ equal to a μ+α*μ^2^, where α is the dispersion parameter [29]. We chose a negative binomial regression instead of a Poisson regression because we found statistical evidence of non-compliance with the assumption of equality between mean and variance after conditioning on the linear combination of covariates. We defined overdispersion when the dispersion parameter α was greater than 0 at a 95% confidence interval. In both cases, we performed two adjusted models. In model A, we adjusted for sex and age; in model B, we adjusted for sex, age, location and type of university, postgraduate studies, and lecturer/professor at a university. We also developed a forest plot as a graphical strategy to represent the disparity measures calculated for the dichotomous outcomes in all the models performed.

We considered the complex sample design (PSU clustering, strata and the unequal selection probabilities of the sampling units) using the *svy* module of the Stata/SE v15.1^®^ statistical software (Stata Corp LP, Texas, USA) [30]. Robust standard errors were estimated via first-order Taylor series linearization [27] and we reported 95% confidence intervals (95% CI). Code to reproduce statistical analysis is available in S5 File.

### Ethical considerations

The open-access dataset (http://iinei.inei.gob.pe/microdatos/) are anonymous. During the implementation of the survey, only those who gave their informed consent were surveyed. The principles for human ethical research outlined under the Declaration of Helsinki were respected at all times [31].

## Results

### Sociodemographic and academic-occupational characteristics by profession

ENSUSALUD-2016 surveyed 2,216 doctors and 2,882 nurses. We did not include 190 doctors and 5 nurses because they graduated from a foreign university. Additionally, we excluded one doctor due to missing data for the location of their university. This analysis included 2,025 doctors and 2,877 nurses. The average age of doctors was 46 years old (S.D.: 12 years) and ranged between 25 and 75 years. The mean age of nurses was 43 years old (S.D.:10.7 years), ranging between 23–76 years. Three-fourths of the doctors were male, while almost all the nurses were female. Independently of their profession, approximately half of the participants belonged to health facilities located in Metropolitan Lima (the capital of Peru). Approximately half of the doctors and nurses graduated from a University located in Lima (Metropolitan area). On the other hand, most doctors but only roughly half of nurses graduated from public universities. The mean age of onset of professional practice was 28 years (S.D.: 4.5 years) and ranged between 16 to 57 years for doctors. In the case of nurses, the mean age was 27 years (S.D.: 5.7 years), ranging between 20–62 years. Only 21% of doctors and 17% of nurses were lecturers/professors at a university, and finally, 43.2% of doctors and 31.3% of nurses held post-graduate degrees. Table 1 summarizes the characteristics of both professional groups.

**Table 1:** General characteristics of doctors and nurses from Peru, 2016.

Characteristics	Doctors		Nurses		p*
	N = 23129	%	N = 34424	%	
**Demographic**					
Mean (SD) age in years	46.1 (12.0)		43.1 (10.7)		0.022¶
Sex					
Male	16495	71.3	2355	6.84	<0.001
Female	6635	28.7	32070	93.2	
Place of residence					
Other regions	9774	42.3	16149	46.9	0.17
Metropolitan Lima	13355	57.7	18276	53.1	
Marital status					
Married/cohabiting	15223	65.8	21426	62.2	0.337
Singleǂ	7906	34.2	12998	37.8	
Living arrangements					
Accompanied	19505	84.3	31098	90.3	<0.001
Alone	3625	15.7	3326	9.66	
**Education**					
University’s location ƚ					
Lima Metropolitan	12473	53.9	15220	44.2	0.032
Other regions	10656	46.1	19204	55.8	
Type of university ƚ					
Private	6957	30	14046	41	0.014
Public	16173	70	20378	59	
**Academic-occupational**					
Mean (SD) age of onset of professional practice	28.0 (4.5)		27.0 (5.7)		0.049¶
Lecturer/professor at University					
No	18167	78.5	29686	86.2	0.011
Yes	4963	21.5	4738	13.8	
Postgraduate studies					
No	13142	56.8	23719	68.9	0.002
Yes	9988	43.2	10705	31.1	

Note: N = wheighted absolute frequency; % = column weighted proportion; SD = standard deviation.

* Chi-squared test design-based (Rao Scott’s correction).

^ƚ^ Numbers do not add up to the total due to missing values.

^ǂ^ Including widowed and divorced.

^¶^ Wald test design-based.

### Disparities in scientific research activity between doctors and nurses

**Fig 1:**
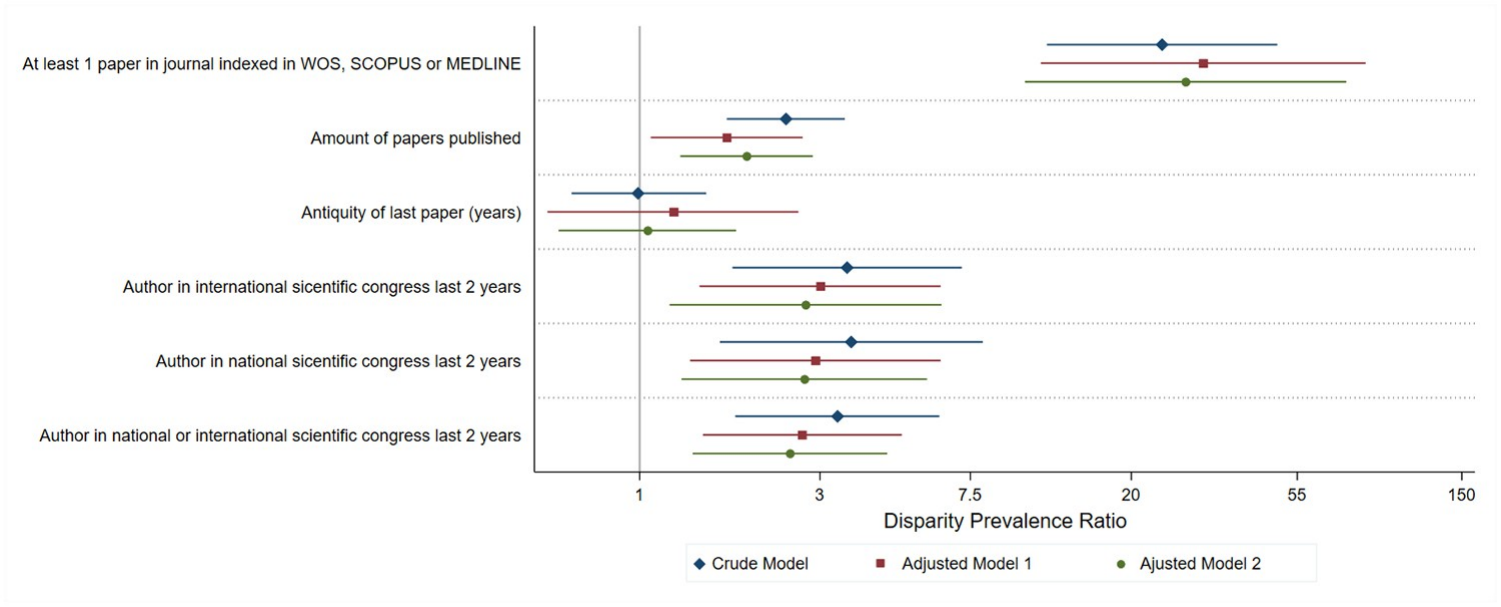
Disparities in scientific activity among doctors and nurses: Crude and adjusted models. Peru, 2016.

We found significant differences in the percentage of doctors (13.9%) and nurses (0.6%) that had published at least one article during their professional practice in an indexed journal (WoS, Scopus or Medline). There was also a disparity in the frequency of participation of doctors versus nurses as authors of a research study in a national or international conference (8.4% vs 2.5%). Table 2 and Fig 1 show the estimates for each of the outcomes studied in the three models constructed.

**Table 2:** Disparities in scientific research activity among doctors and nurses from Peru, 2016.

Outcomes	Doctors		Nurses		Comparisons: Doctors vs Nurses (reference)								
					Crude model			Adjusted Model A			Adjusted Model B		
	N = 23129	%	N = 34424	%	cDPR*	95% CI	p	aDPR*	95% CI	p	aDPR*	95% CI	p
Original article published in journal (WoS, Scopus or Medline)													
At least one research paper	3218	13.9	198	0.6	24.15	11.97–48.72	<0.001	31.01	11.52–83.47	<0.001	27.86	10.46–74.19	<0.001
Mean (SD) number of papers published‡	3.2 (5.6)		1.3 (0.6)		2.44ƚ	1.70–3.49	<0.001	1.70ƚ	1.07–2.69	0.026	1.92ƚ	1.28–2.87	0.002
Mean (SD) time last paper was published in years‡	5.3 (4.9)		5.3 (5.1)		1.01ƚ	0.64–1.59	0.977	1.31ƚ	0.59–2.89	0.499	1.08ƚ	0.58–2.01	0.814
Participation as author in a conference within the last 2 years													
International	521	2.3	219	0.6	3.55	1.76–7.13	<0.001	3.01	1.45–6.26	0.003	2.76	1.21–6.30	0.016
National	1579	6.8	645	1.9	3.64	1.64–8.10	0.002	2.92	1.36–6.26	0.006	2.73	1.29–5.77	0.009
Any type (international or national)	1940	8.4	864	2.5	3.34	1.80–6.22	<0.001	2.7	1.47–4.94	0.002	2.51	1.39–4.53	0.003

Note: WoS = Web of Science; N = wheighted absolute frequency; % = wheighted proportion; CI = confidence interval; cDPR = crude Disparity Prevalence Ratio; aDPR = adjusted Disparity Prevalence Ratio; SD = standard deviation.

* Unless otherwise indicated, DPRs are reported and calculated using generalized linear regression models with Poisson family error and log link function accounting for complex survey design.

^ƚ^ Disparity Mean ratios are reported and calculated using negative binomial regression accounting for complex survey design with mean μ and variance function of Cameron & Trivedi (NB2) equal to μ+αμ2, where α is the dispersion parameter. A paremeter α different of 0 is evidence against the assumption of media = variance of Poisson regression. Outcome number of papers published: αcrude model = 0.59 (95% CI: 0.28–1.26); αmodel A = 0.46 (95% CI: 0.23–0.93); αmodel B = 0.37 (95% CI: 0.19–0.73). Outcome time last paper was published in years: αcrude model = 0.85 (95% CI: 0.73–0.99); αmodel A = 0.73 (95% CI: 0.56–0.95); αmodel B = 0.70 (95% CI: 0.51–0.96).

^‡^ analysis only includes doctors and nurses who refered that they published in journal indexed in WoS, Scopus or Medline.

Model A, adjusted by sex and age (years).

Model B, adjusted by sex, age (years), university´s location, type of university, postgraduate studies, and being a lecturer/professor at university.

In the crude models, we observed that the proportion of doctors with at least one published scientific article in an indexed journal was greater than the proportion among nurses (cDPR = 24.15; p<0.001). Compared to nurses, doctors had a greater average number of articles published (cDMR = 2.44; p<0.001), and a greater proportion of participation as an author in national (cDPR = 3.64; p = 0.002), international (cDPR = 3.55; p<0.001) or any of these conferences (cDPR = 3.34; p<0.001).

In the adjusted models, doctors were more likely to have published an article in an indexed journal (aDPR = 27.86; p<0.001); and had a larger quantity of articles published (aDMR = 1.92; p = 0.002). Likewise, doctors compared to nurses were more likely to have authored a national or international conference abstract (aDPR = 2.51; p = 0.003). Conversely, we did not observe any significant differences between doctors and nurses regarding the time from the publication of their last article (aDPR = 1.08; p = 0.0814).

### Discussion

#### Main findings

Our findings show significant disparities between doctors and nurses working in health facilities in the Peruvian Health Care System. While the evidence suggests that the development of scientific research in the health care workforce in Peru is deficient [32]; our results show that these deficiencies are much more significant among nurses than among doctors. These disparities are more significant in the publication of papers in peer-reviewed journals than in the presentation of research at conferences. Given that we have not been able to find similar studies conducted elsewhere, we cannot know if this is a prevalent phenomenon in other countries. However, we have to consider that different working, training and incentive conditions of both professional groups in other middle to low-income countries, and even in developed countries, would lead to a similar phenomenon, although perhaps with less apparent disparities [4,12,13].

#### Factors involved and plausibility

Sex, age, graduating from a university located in the capital city, having post-graduate studies or being a university professor are all factors that might increase the opportunities to carry out scientific research; therefore, the disparities found could be due to the unequal distribution of these variables between doctors and nurses. The fact that these disparities persist (and in similar magnitude) after adjusting for the factors mentioned above strongly suggests that other factors related to the profession and work environment would limit the chances for nurses to develop a proper level of scientific research activity. These disparities not only represent a problem of inequality of opportunities for personal and skills development but could also have repercussions on the health of the Peruvian population since scientific evidence needs to be generated and disseminated at the same time in all areas of the Health Care System so that it can be articulated promptly and used to improve the health of the population. Likewise, these disparities may represent an unintended result of the higher education curricula, as it is very different in both professions. Our study is one of the first to identify essential disparities in scientific research activities of healthcare professionals in a developing Latin American country and could be used as a baseline for further interventions aimed at improving the opportunities for scientific research activities in healthcare systems.

Our results reflect that both doctors and nurses have little involvement in research. We have seen a similar condition in some countries in Europe, Asia and Oceania [33–35], where the total number of doctors who have published scientific articles is less than one-fourth of the total. In Peru, the situation is even worse since 6.8% and 4.6% of doctors that work in hospitals have published at least one article in SciELO and Scopus scientific journals, respectively [36]. Another study found that the publication of scientific research in indexed journals is an infrequent practice among doctors with masters or doctoral degrees in Peru. Unfortunately, this reinforces the fact that research on doctors remains scarce even in the subgroups that have been trained in these matters [37]. Concerning nurses, there has been more significant participation in scientific research in developed countries [38,39]. A study conducted in Malaysia found that 4.5% of nurses had published at least one scientific article, which differs from our results by only 0.5%. Beyond the lack of scientific production of nurses and doctors, there is a clear disparity in the scientific research between them, with a 27 to 1 ratio between medical and nursing professionals in Peru. This scenario differs significantly in Malaysia, where the ratio is approximately 5 to 1 [35,40].

The disparities observed are multifactorial and complex. Beyond the disproportionate distribution of each factor between both professional groups, it is crucial to consider that each factor can exert a different effect on each group. Although we do not have the data available to identify such factors, we will preliminarily develop some hypotheses that should be evaluated in further studies. We believe that multiple factors can coexist, and they range from inadequate research training during undergraduate studies to work overload in the clinical care setting. Notwithstanding, the lack of scientific production, which is far worse among nurses, there is a lack of studies addressing this phenomenon. Nurses in Peru have expressed low motivation levels for scientific research due to a lack of economic incentives or proper professional recognition [41,42]. Meanwhile, nurses dedicated to caregiving have expressed an unfavourable perception of scientific research claiming that they do not consider it part of their professional responsibilities and consider that the additional workload does not favour their development [43].

There might also be contrasting incentives to conduct research, even without a salary. Some doctors see this as a recognition of professional development. Likewise, doctors are more prone to carry out essentially positivist biomedical research than the socio-phenomenological research approach that is more frequently taken by nurses [44,45]. Due to a more excellent scientific "value" (erroneously perceived by the wider scientific community) and the broader visibility of the positivist approach compared to the socio-phenomenological approach, it would be easier for doctors to disseminate their results research. Another key factor could be the difference in the access to research funding or the facilities to conduct *ad honorem* (non-profit) scientific research that doctors would have since they earn a higher income [46]. However, these are only hypotheses. We reiterate that the barriers that could explain these disparities are plentiful, and we need further evidence to identify the main factors that could lead to low scientific research activity in both professionals.

Some educational factors may be responsible for the gap reported in our study. Even though the research methods training is inadequate in almost all Peruvian medical faculties, the situation of nursing faculties maybe even worse. There is a generalized perception of a deficiency in research training in undergraduate programs in Peru [47,48]. Time devoted to training is lacking. Only 1.75% of the total coursework corresponds to research training, and these courses do not focus on aspects related to publication [49]. Furthermore, the quality of research training provided by the universities is flawed, as can be inferred when taking into account that most of the professors who teach or advise these subjects do not publish scientific articles [50,51]. In addition, the deans of the faculties of health sciences also lack scientific publications [52]. Herein relies on the importance of fostering academic activities, which are indeed more common among doctors. Although we have not found studies that evaluate research training in nursing students in Peru, given our results, we believe that their situation is even worse than that of medical students. It is worth pointing out that a medical degree lasts seven years, whereas nursing lasts only five years in Peru.

Among the structural factors of professional life, the workload in the Peruvian Health Care System would greatly hinder the performance of scientific research ability in both professional groups [53,54]. Although the present study did not evaluate whether this is an associated factor with low scientific research productivity, limited time is a widely recognized limiting factor in other aspects of daily life [53,54]. This situation is aggravated in Peru because scientific research lacks proper incentive for doctors and nurses alike: scientific publication weighs little to nothing in a *curriculum vitae* to compete for medical or nursing specializations [55], and almost no scientific society requires publications as a prerequisite for membership [56]. To make matters worse, in many cases, dedication to scientific research will negatively affect a professional’s economic income, especially when there is no form of adequate professional recognition for research conducted [57]. Future studies should identify potential structural factors that can be modified and play an essential role in low scientific research productivity in both groups of professionals.

#### Limitations and strengths

Our analysis has some limitations. First, the data surrounding scientific production and participation in conferences corresponds to the self-report from the participants. This could lead to a risk of measurement bias (*recall bias*). The surveyed professionals might not remember if they have published an article or not in their lifetime or their participation in a scientific conference in the last two years. Nevertheless, we reduced the risk of inaccurate recall of a publication by a posterior verification of the journal and database specified by the participant. On the contrary, there may be a greater risk of measurement bias in the number of publications in WoS, Scopus or Medline, which may underestimate non-differential misclassification disparities. Likewise, there is a slight chance that due to social desirability bias, one of the professional groups would systematically and incorrectly report participation in non-existent conferences or journal publications (or a greater number of them), which would skew the disparities found in a progression without adequate foundation. However, we consider it a rare or isolated event; therefore, we could overlook its impact on the overall estimates. Second, as secondary data analysis, essential variables were not considered: university curriculum, personal and professional interest in scientific investigation, undergraduate training courses, and incentives for research within the workplace. Nevertheless, the analysis methods employed allow for significant variables among the participants and estimate disparities that could be explained by structural differences in the Peruvian Health Care System that should be investigated more thoroughly. Third, the results of our study are based on a national representative sample of doctors and nurses within health facilities in 2016. While our results apply to a large percentage of the health workforce in Peru, there have been no fundamental changes in health research policies in the latest years in Peru; therefore, we believe our results remain valid.

Beyond the limitations expressed, our study has an adequate national representative sample of both groups of health professionals understudy, which allows us to conclude that doctors and nurses in Peru publish in indexed scientific journals and participate in conferences. However, the frequency of research and publication is still low compared to other developed countries [58]. Governmental support is indispensable to promote scientific health research at all levels of the health care system, such as nationally competitive research funding and incentives and strategic planning focused on the production of health sciences research [59]. We anticipate that the increase in scientific research participation leads to growth in the health sciences and society with the generation of primary scientific evidence, which will create more informed decision-making on behalf of the government [60,61]. Systematic barriers to the increase in scientific research activity could be overcome if the administrative policies within the Peruvian Health Care System are modified, and research opportunities include the impact of the prosperity of health care professionals, including home life, clinical practice and organizational culture [5]. For example, the implementation of support units for nursing research in hospitals has been shown to have a great effect on the increment of scientific production of both nurses and hospitals as a whole(57). If replicated nationwide, this observation could significantly increase scientific research production around the country for nursing professionals. We believe that the implementation of public policies should be based on the best evidence available to provide the best opportunity for its success and include the professionals’ direct participation.

#### Implications, recommendations, and future studies

Our study can serve as a meaningful foundation that stems from the continual effort that many national organizations have struggled to promote in scientific research on health sciences. For this reason, periodic cross-sectional studies are suggested to monitor progress in the increase of scientific research activity among doctors and nurses and reduce existing disparities among professionals. These studies should measure scientific research activity and consider other limiting variables to avoid the information limitations in this valuable line of work. It would be interesting to complete these studies in various countries to get a more comprehensive grasp of these disparities on an international level, including the correlation between the economic income of professionals and scientific output, and the designation of GDP to research, development, and innovation.

We believe that scientific research activity among doctors and nurses is a binding obligation in Peru so that the ability to develop primary evidence can be utilized to inform decision-making in the health care system. However, professionals currently face multiple barriers to the successful development and execution of scientific research and, what is more concerning, these barriers are systematically unequal and disproportionally affect one profession over the other. The formulation and implementation of multisector public policies that endorse scientific research and investigation by Peruvian health care professionals should equitably encourage the advancement of research among all health care professionals.

#### Conclusion

In conclusion, there are disparities in self-reported scientific production, which is higher among doctors in comparison with nurses working in the Peruvian health system. Disparities are much more significant for article publication than for authoring in conference abstracts.

## Supporting information

S1 File R script for converting dataset from SPSS format to Stata v14 format. This code converts data in SPSS format («*.sav») to Stata v14 format («*.dta») and generates a raw data named S1 Dataset.
(R) Click here for additional data file.

S2 File Raw dataset. (DTA)

S3 File Do file for preparing raw dataset into tidy dataset for analyzing. This code converts raw dataset named S1 File in tidy dataset, S4 File.
(DO) Click here for additional data file.

S4 File Tidy dataset. (DTA)

S5 File Do file to analyze tidy dataset. This code does all statistical analysis reported in the paper.
(DO) Click here for additional data file.
